# Encouraging Patients to Ask Questions: Development and Pilot Testing of a Question Prompt List for Patients Undergoing a Biopsy for Suspected Prostate Cancer

**DOI:** 10.3390/curroncol30020162

**Published:** 2023-02-08

**Authors:** Orlando Rincones, Allan ‘Ben’ Smith, Peter Chong, Pascal Mancuso, Verena Shuwen Wu, Mark Sidhom, Karen Wong, Diana Ngo, Paul Gassner, Afaf Girgis

**Affiliations:** 1 Ingham Institute for Applied Medical Research, Liverpool 2170, Australia; 2 South West Sydney Clinical Campuses, UNSW Medicine & Health, University of New South Wales Sydney, Liverpool 2170, Australia; 3 Lake Macquarie Urology, Newcastle 2290, Australia; 4 Liverpool Cancer Therapy Centre, South Western Sydney Local Health District, Liverpool 2170, Australia; 5 Department of Urological Surgery, South Western Sydney Local Health District, Liverpool 2170, Australia

**Keywords:** prostate biopsy, prostate cancer, question prompt list, treatment decision-making

## Abstract

This study assessed the acceptability and feasibility of a question prompt list (QPL) to facilitate informed treatment decision-making in men with suspected localised prostate cancer, which involves values-based choices between options with similar efficacy but different side effects. The QPL was developed through iterative consultation with consumers, clinicians and researchers. Acceptability was assessed using study-specific questions regarding QPL satisfaction and usefulness and qualitative interviews. Feasibility was determined via the proportion of men given the QPL according to medical records and the completion of standardised measures of decisional outcomes. Quantitative data were analysed using descriptive and univariate statistics. Qualitative data were thematically analysed. Fifty-two men consented; 34 provided data for analysis. The QPL recipients reported moderate–high content satisfaction (70.6%) and perceived usefulness in guiding appointments when receiving biopsy results (64.7%). Two main qualitative themes also indicated the QPL acceptability: (1) the freedom to ask—acceptable timing, flexible usage and usefulness of the QPL, and (2) satisfaction with the QPL content. However, only 18.4% of eligible men received the QPL, indicating limited feasibility. The QPL is safe and acceptable, but further research is needed regarding how to facilitate the uptake of the question prompt list in clinical practice.

## 1. Introduction

Prostate cancer (PC) is the second most commonly diagnosed cancer in men worldwide, with approximately 1,414,259 new PC cases diagnosed in 2020 [1]. PC diagnosis is confirmed with a prostate biopsy [2], with more than one million prostate biopsies performed yearly in the United States [3]. Waiting for biopsy results can be challenging for patients with suspected cancer, who tend to experience increased anxiety during this time [4,5]. This wait might trigger questions and concerns about the biopsy results surrounding prognosis and treatment options.

Decision-making in men diagnosed with localised prostate cancer (LPC) has been widely studied, as no gold-standard treatment exists. Treatment options for LPC include active surveillance, radical prostatectomy (open or robotic-assisted) or radiotherapy [6]. Each treatment has different side effects, which can be experienced for many years post-treatment [7,8]. Research also shows that men with LPC might experience decisional difficulty and regret [9]. Our previous research revealed that 65.9% of men with LPC experienced moderate-to-high levels of decisional difficulty after receiving their diagnosis [10], most men made a treatment decision immediately after diagnosis and some of them were unaware of radiotherapy as a treatment option [11]. For these reasons, all available treatment options should be offered (as stated in current guidelines [6]) and discussed immediately after diagnosis. Several decision aids were developed to improve decisional outcomes in men weighing up treatment options for LPC in light of their values and preferences so they can effectively engage in shared decision-making with their doctors. While many of these decision aids are effective in improving LPC treatment decision-making, their widespread use in clinical practice has not been achieved [12].

Question prompt lists (QPLs) have been utilised in broader cancer populations to facilitate communication with the cancer care team [13,14,15] and found to have the potential to facilitate treatment decision-making discussions [13]. A recent review found that QPLs help patients ask more questions (particularly difficult questions), have the potential to reduce follow-up anxiety and might be more helpful than standard information materials [16]. However, QPLs’ impact on oncology decision-making is still unknown [17]. QPLs might facilitate the discussion of treatment options and enable patients to be more informed and prepared for their treatment decision-making. This study aimed to develop a QPL for men undergoing a prostate biopsy and assess its acceptability and feasibility for implementation in routine care. This feasibility study will allow us to further evaluate the role of QPLs in prostate cancer decision-making.

## 2. Materials and Methods

### 2.1. Study Design, Participants and Procedures

This was a phase I pilot study that included two stages. First, we developed a study-specific QPL for men with suspected LPC (November 2018 to May 2019). The questions were derived from QPLs for cancer patients more generally [18]; our previous research in this area [10,11]; a review of the scientific literature [13,15]; existing resources for men with LPC (from the American Cancer Society, Macmillan Cancer Support, Prostate Cancer Foundation of Australia and Cancer Council of Australia); and input from a multidisciplinary team that included consumers, urologists, radiation oncologists, nurses and psycho-oncology researchers. The second stage (from June 2019 to May 2020) involved the pilot testing of the QPL. The QPL was provided to men after they had a prostate biopsy to facilitate discussion at their subsequent consultation. Men were offered the choice of a printed or online QPL (with the option to download and print if needed).

**Figure 1:**
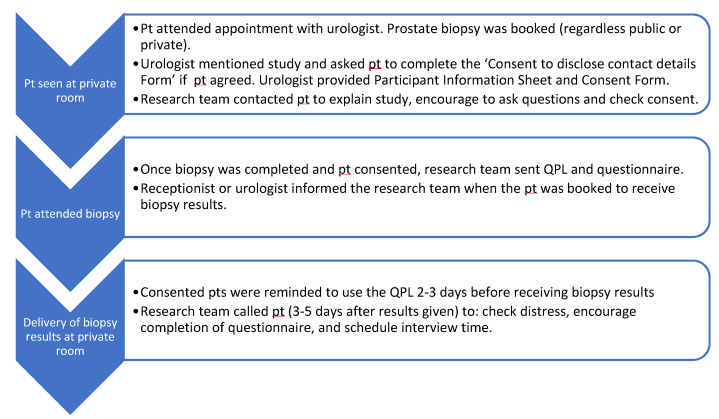
Study procedure. Pt, patient; QPL, question prompt list.

The target population was men aged 70 years or less with prostate-specific antigen (PSA) < 50 and having an initial biopsy for suspected LPC at private urology clinics in South Western Sydney (Macarthur Urology), Newcastle (Lake Macquarie Urology) or the public Liverpool Hospital Transrectal Ultrasound Scan (TRUS) biopsy clinic. Men diagnosed with advanced prostate cancer were excluded. Given the focus on QPL acceptability/feasibility rather than efficacy, a sample of 30 patients was deemed sufficient [19]. Recruiting urologists were also invited to complete an interview exploring their perspectives on using the QPL. Figure 1 outlines the study procedure.

The participants completed a questionnaire that assessed sociodemographic details, as well as usage and satisfaction with various aspects of the QPL, including its content, length and format. Participants were also invited to take part in an optional semi-structured interview that explored barriers and enablers to QPL usage in greater depth. Urologists completed a brief semi-structured interview once patient data collection was finalised to determine their satisfaction with the QPL, its impact on their practice and barriers/enablers to continued QPL use.

### 2.2. Outcome Measures

*QPL feasibility, acceptability and usage*—Feasibility was assessed via the proportion of patients who received the QPL at participating recruitment sites. Acceptability was evaluated via the satisfaction and usefulness items in the questionnaire (mean scores). Usage was assessed via the average scores in the usability section of the questionnaire (e.g., how much time did you spend looking at the QPL?).

*Decision-making outcomes*—The questionnaire also included the following measures:

- The single-item Control Preferences Scale [20];
- The 16-item Decisional Conflict Scale [21];
- The Patient Satisfaction Questionnaire (PSQ) [22,23].

These scales were validated and widely used in prostate cancer decision-making research [24]. These outcomes helped to describe the decision-making process and outcomes experienced by men, but this study was not sufficiently powered to make any definitive conclusions regarding the impact of the QPL on decision-making. These outcomes were included to assess the feasibility of collecting such data for a potential future study of QPL effectiveness if acceptability and feasibility were demonstrated.

### 2.3. Analysis

Given the focus on QPL acceptability/feasibility rather than efficacy, quantitative data analysis was primarily descriptive, including the use of frequencies, proportions, means and standard deviations. We also used the independent *t*-test and Fisher’s exact test when appropriate. Qualitative analyses comprised thematic analysis [25] of verbatim transcriptions of semi-structured patient interviews to examine QPL usage and satisfaction and to identify common barriers and enablers for its use. The study team developed the interview schedule (Appendix A). One researcher conducted the interviews (O.R.), and two researchers (O.R. and A.B.S.) familiarised themselves with the data, coded the transcripts, grouped codes into potential themes and identified emerging themes after multiple discussions. Discrepancies were resolved through discussion and consensus. Data saturation (the stage where enough data has been collected to understand the phenomenon under study and any further data collection does not add any value or further insights) was reached after ten interviews.

### 2.4. Ethics

The study was approved by the South Western Sydney Local Health District Human Research Ethics Committee (HREC/18/LPOOL/470). All study participants provided signed informed consent. Ethics, data privacy and confidentiality followed the principles of the Declaration of Helsinki [26] and the standards of the Australian Code for the Responsible Conduct of Research [27].

## 3. Results

### 3.1. Stage 1—Question Prompt List Development

A two-step approach was used to develop the QPL. The first step included reviewing QPLs for cancer patients more generally [2]; our previous research in this area [10,11]; the scientific literature [13,15,28,29]; and existing resources for men with LPC, including the Prostate Cancer Foundation of Australia, Cancer Council Australia, American Cancer Society and Macmillan Cancer Support websites. This resulted in 211 potential questions, some of which were repeated across multiple sources (e.g., what are the side effects of each treatment?). Authors A.B.S. and O.R. reviewed the questions and excluded duplicates, considerably similar questions and questions that were not applicable (i.e., questions regarding advanced prostate cancer). This resulted in a 58 questions being short-listed.

The second step involved an iterative process of receiving feedback on the 58 questions from a multidisciplinary team, including consumers (n = 3), urologists (n = 3), radiation oncologists (n = 2), nurses (n = 1) and psycho-oncology researchers (n = 1). The multidisciplinary team agreed on a QPL with 26 questions, a space for patients to write their main concerns and to add further questions they may have. The questions were presented in a half-fold A4 brochure (Appendix B—Figure A1) at a Liverpool Hospital Urology team meeting, which included nine urology specialists (six of whom had never seen the QPL before) who provided further feedback. The urology team agreed with the 26 questions but suggested adding a supplement with three key questions for patients not diagnosed with prostate cancer (Appendix B—Figure A1). This suggestion was accepted by the broader research team.

### 3.2. Stage 2—QPL Pilot Testing

#### 3.2.1. Participant Characteristics

**Figure 2:**
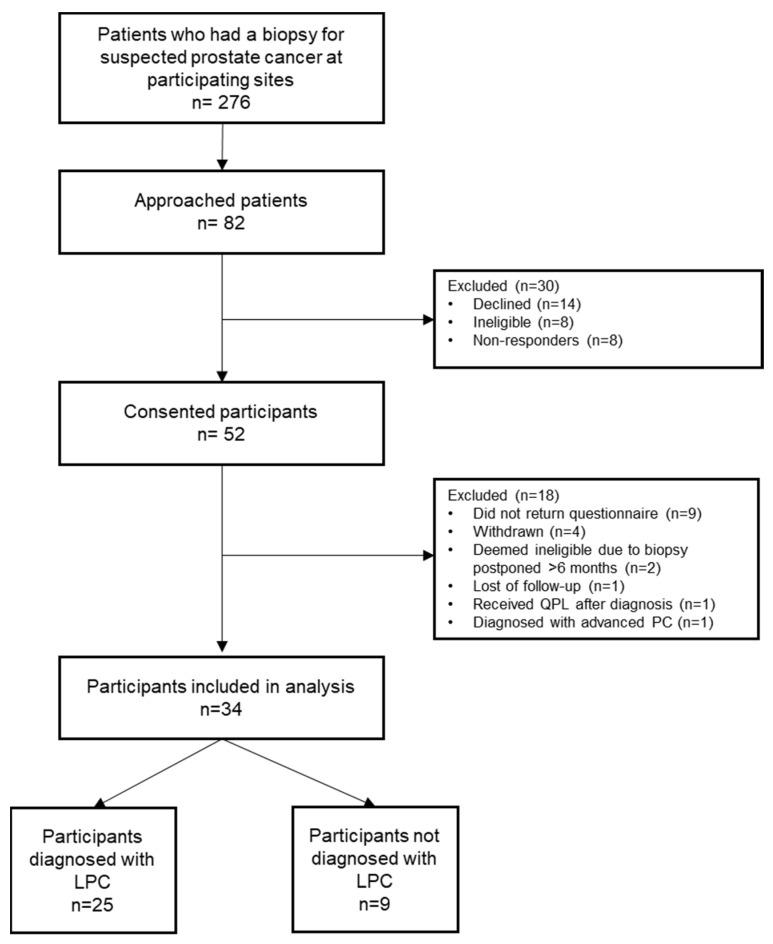
CONSORT diagram. QPL, question prompt list; LPC, localised prostate cancer.

Eighty-two patients were invited to participate in the study, of whom 52 (63.4%) consented. Eighteen participants (34.6%) were excluded (see Figure 2 for reasons). As a result, data for 34 participants (65.4%) were included in the analyses. Study results are presented for the entire sample and the two biopsy outcomes (diagnosed with LPC, n = 25; not diagnosed with LPC, n = 9). On average, participants were 64.6 years of age (range 43–78). Nearly 65% of the sample completed post-school vocational studies or university degrees, and most were retired (44.1%) or full-time employed (41.2%). Most participants were married (82.4%), born in Australia (79.4%) and had no history of cancer (85.3%) (see Table 1).

#### 3.2.2. Quantitative Findings

Feasibility and QPL usage: Out of 276 patients who had a biopsy for suspected localised prostate cancer at participating sites, 29.7% were offered study participation and 18.4% were given the QPL (see Figure 2). Most participants preferred the printed version of the QPL (82.4%, see Table 2) and read it before receiving the biopsy results (76.5%). The average time looking at the QPL was 18 min, it was generally reviewed individually (55.9%) and caused distress to only one patient (3%). On average, seven questions were selected from the QPL before the consultation, but only five were asked during the appointment. Half of the participants took the QPL to the consultation and 41.2% referred to it during the discussions with their doctor. Participants diagnosed with LPC asked significantly more questions than those without LPC (*p* = 0.02, t = 1.42). See Table 2 for more details.

QPL usefulness and satisfaction: Most participants indicated moderate-to-high levels of QPL usefulness in guiding their appointment when receiving biopsy results (64.7%), helping them to understand their diagnosis (61.8%) and treatment options (58.9%). Additionally, most participants were satisfied with the contents (70.6%), layout (67.6%) and length (67.6%). No significant differences were found between those diagnosed with LPC and those not diagnosed (see Table 3).

Selected questions: Only 14 of 34 participants provided details about which questions they selected (Appendix C—Table A1). The remaining participants stated they had not kept the QPL and could not remember the questions chosen when followed up after the consultation to discuss the biopsy results. The most frequently selected questions were “Has the cancer spread outside my prostate and how fast is it growing?” (76.9%) and “What are the side effects of each treatment? Both short-term and long-term” (76.9%), followed by “What treatment do you recommend and why?” (61.5%) and “How long is it likely to take for me to recover after each treatment?” (61.5%). Other questions such as “Will I have urinary problems (like incontinence)? Will I have to wear pads?” and “How will treatment affect my quality of life?” were selected by more than half (53.8%) of participants. Each of the 26 questions was selected at least once by participants.

Decisional outcomes and satisfaction with the consultation: The most preferred treatment was radiotherapy (36%), followed by robotic prostatectomy (24%). Fifty-two per cent of the participants preferred high control over their decision-making, while 28% preferred a shared approach and only 12% opted for low control. Moreover, the average decisional conflict total score was 22.2 (SD = 16.2), and the subscales ranged from 17.7 to 27.4 (see Table 4). Regarding clinical thresholds for the Decisional Conflict Scale, 72% of participants did not experience decisional difficulty (scores < 25), 8% had moderate difficulty (scores > 25–37.4) and 16% reported high difficulty (scores > 37.5). Finally, the Patient Satisfaction Questionnaire revealed an average score of 84.9 (SD = 19.4; range: 34–100), with no statistical difference between those with or without LPC (*p* = 0.806, t = 0.312).

#### 3.2.3. Qualitative Findings

**Figure 3:**
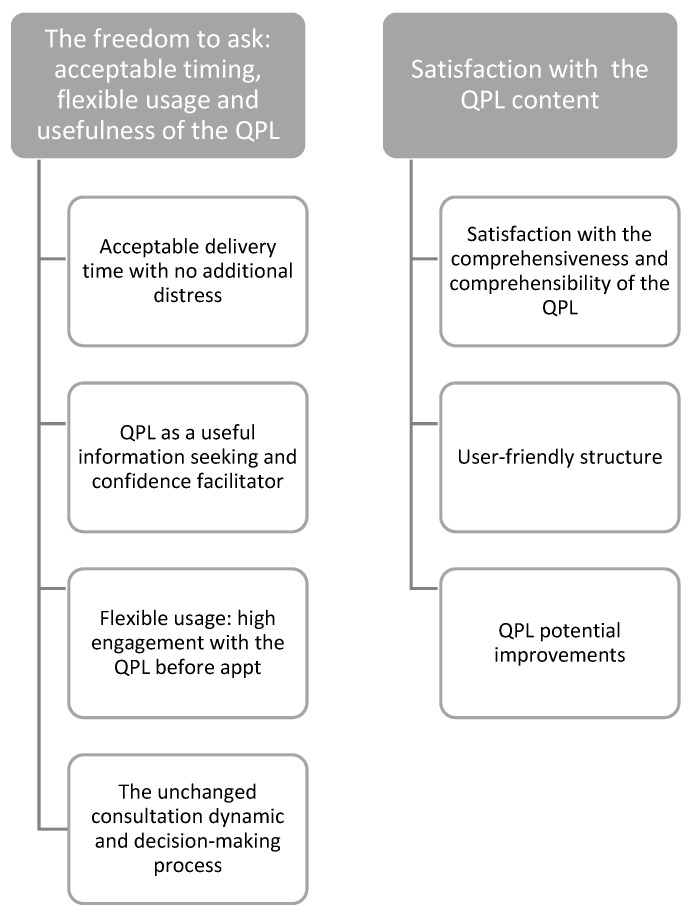
Thematic map.

Two main themes were identified, as depicted in Figure 3. Subthemes and quotes are detailed in the Supplementary Material—Table S1.

The freedom to ask—acceptable timing, flexible usage and usefulness of the QPL: Participants believed the QPL should be delivered before receiving biopsy results and did not trigger additional distress. Some expressed other appropriate times, including pre-biopsy or post-diagnosis.

*I received it and thought, “Well, I’ll read through this and it might be of some help for me,” and I was okay with it… I thought it was at a good time because having first spoken with the doctor and then going for the results from the biopsy, the questions on there would be what I would be interested in having answers for.*

P004

Participants reported different approaches when using the QPL at different time points. Most of them used the QPL before receiving their biopsy results. Some used the QPL during the appointment with their doctor, while others did not.

*I read it [the QPL] as soon as I received it. And then I read it again before I went to see him to refresh my memory. And I took it with me and referred to some of the questions during the course of the consultation.*

P026

The QPL was a helpful tool for participants waiting for biopsy results. The QPL aided in identifying and reminding men of the key questions or concerns they had before the consultation. Some found that the QPL contained questions they already had, reinforcing their need to ask them. Others found relevant questions they had not thought of. This process of reiterating or discovering new questions to ask their specialists prompted them to seek information in advance, making participants feel reassured or at ease.

*So, I think it’s a pretty useful tool to have even though it’s only the first initial little flyer thing to ask the doctor questions. I think it’s good because your mind, it can be thinking of other things. There’s a lot of things that you can forget to ask him. So, I think it’s a good system. I think it’s a good thing to have, you know, to be able to bring something home and go through it rather than just have nothing at all.*

P003

The QPL did not significantly impact the overall consultation dynamic and decision-making process. All participants agreed they would recommend the QPL to a friend.

Satisfaction with the QPL content: The number of questions was perceived as acceptable and easy to understand without difficult medical terminology.

*I thought the number of questions was fine. It wasn’t too long a read.*

P026

*I think it’s laid out in a pretty good manner, really. It’s easy to understand. It’s quite clear and the questions are quite clear… I think you got it pretty well covered.*

P004

The structure and layout of the QPL were also seen positively.

*I like the fact that it was just a folded up small page, easy to handle. The font I found easy to read… The colour was pleasing on the eye. It wasn’t outstanding or bright and everything like that. It was subtle.*

P026

Some participants provided suggestions for improvement.

*…adding certain sites that you recommend in your flyer to say the Australian Prostate Cancer Organisation website is probably the more reputable site to look up information. So, that may be something you put in the flyer. You have links to—or web addresses to reputable information sources.*

P016

## 4. Discussion

A QPL specifically for men with suspected LPC (Appendix B—Figure A1) was successfully developed after rigorous and iterative consultation with consumers, urologists, radiation oncologists, nurses and psycho-oncology researchers. The QPL was then piloted among 34 men who were found to be satisfied with the tool and found it useful based on quantitative and qualitative feedback. However, the feasibility of the QPL was limited, as only 29.7% were offered the option to receive the QPL as part of the study and 18.4% were given the QPL. The limited QPL feasibility might have partly been due to “additional admin work” reported by clinicians, as a member of the care team had to explain the study and provide the study information package and link to the research team. Provision of the QPL as part of the standard information provided to the patient may overcome this issue, as the “extra work” was largely associated with the QPL evaluation rather than the QPL itself.

The QPL and related study materials were only available in English, which may have further limited the feasibility, as one of the recruitment sites (Liverpool Hospital) was located in a culturally diverse area where 49% of the population speak a language other than English at home. The brevity and simplicity of the QPL may aid translation and further evaluation of the QPL in men from culturally and linguistically diverse backgrounds in the future. Urologists may also have been reluctant to give the QPL to men they perceived to be particularly anxious or may have worried that the QPL would adversely impact the doctor–patient relationship by making consultations more formulaic and less personal, although this was not mentioned in the feedback from either the urologists or patients. Further research is needed to better understand both patient and clinician barriers to QPL provision.

The printed version of the QPL was the most popular choice, with more than 82% preferring this option. This might be because the QPL is a relatively small brochure that can be carried easily. Another explanation for printed-version preference could be the age group of our sample (mean age = 64.6), who might feel more comfortable with paper-based resources. Having different formats available is critical in QPL implementation, as there is evidence that format availability (paper-based and online) aids QPL usage in routine care [15]. On average, the men spent approximately 18 min looking at the QPL, suggesting they took their time to consider each question thoroughly. This is supported by qualitative findings of the men who looked at the QPL for up to an hour and multiple times before the consultation, or those who used it more systematically after receiving the diagnosis when they attended a second consultation to discuss treatment options in more detail. These findings reflect high engagement with the QPL. Seventy-six percent of the participants reported using the QPL, which was higher than the 60% found in a study in adult cancer patients (mostly breast and prostate cancer patients) going through curative or palliative radiation therapy [14] but lower than a study in adult advanced or metastatic head and neck cancer patients (stage III or IV) (90%) [30].

On average, participants selected more questions from the QPL before the consultation than what they asked during the consultation. There were moderate levels of usage during the consultation, as only half of the participants took the QPL with them, and 41% of participants referred to it during the discussions with their doctor (higher than in previous studies [30]). Reasons for this might include being shocked by the diagnosis, forgetting to use the QPL, not bringing the QPL to the consultation or not being diagnosed with LPC. Interviews also showed urologists often spontaneously addressed many of the patients’ questions during consultations, removing the need to ask some questions. The interviews showed that the QPL did not substantially impact the consultation dynamics. Although only 41% of men referred to the QPL during the consultation, 64% believed that the QPL helped them guide the appointment. As expected, the men who were ultimately diagnosed with LPC asked significantly more questions than those who did not, which is reasonable as the brochure for those without LPC had fewer questions. These findings were consistent with the outcomes of a systematic review [31] that found that the QPLs were useful in improving patient participation in cancer appointments and made participants ask more questions.

QPL usage before the appointment varied. Most participants looked at the QPL as soon as they received it, suggesting high interest in the resource. In some instances, the QPL became a social resource, as the men discussed it with a relative (generally a partner), reflecting literature highlighting the importance of social support in the PC journey [32], particularly from partners [33]. The number of questions picked before the appointment ranged from none to “most” of the questions, demonstrating individual differences in information needs. The most frequently selected questions (“Has the cancer spread outside my prostate and how fast is it growing?” and “What are the side effects of each treatment? Short-term and long-term”) showed that potential cancer spread and treatment side effects were the key significant topics for patients; hence, clinicians must make sure they clearly cover these topics during consultation.

One of the most critical issues assessed in this pilot study was the potential of the QPL to trigger additional distress. Most participants (97%) did not feel distressed by using the QPL before receiving their biopsy results, and interviews revealed that some men felt “at ease” due to the QPL use, consistent with previous literature [30]. Similarly, an educational tool for men with suspected LPC [34] did not increase anxiety before the appointment to receive biopsy results (anxiety levels were reduced but not significantly). The lack of additional distress may be explained by expecting a negative biopsy result (meaning no presence of cancer) and participants’ coping mechanisms. Moreover, participants commented that the delivery time was appropriate. Some argued that the QPL could be delivered earlier (i.e., before the biopsy), as noted in other studies [14].

Participants reported satisfaction with the QPL content, length, structure and layout. Furthermore, 61% indicated an improved understanding of the diagnosis, and 58% noted the QPL helped them understand treatment options. Usefulness in different domains (around 60%) in this study was slightly higher than in previous studies [14] and lower than in others [30]. Notwithstanding, interviews found that the QPL did not directly influence participants’ treatment decisions or decision-making processes, which was in line with systematic review findings [17]. This is significant because it was not expected to encourage men to select a treatment over the other, but instead inform participants about all available options as an interviewee stated “I didn’t know I had any [treatment] options, mate… I just thought that whatever the doctor said I would have to accept that”. Participants reported multiple benefits from the QPL, including identifying new and relevant questions they wanted to ask their urologist, and prompting patients to look for information in advance. All these benefits explain why all interviewees agreed they would recommend the QPL to a friend in the future. An explanation for this could be the relatively low number of questions (23), which is at the lower end of the range reported in a systematic review (11 to 189) [31].

Interestingly, the preferred treatment option was radiotherapy (36%), as opposed to prostatectomy (either open or robotic), which was reported to be the most popular treatment in previous literature [24,35]. This might have been due to individual preferences, clinical factors, urologist recommendation or the average age of the sample (64 years old). Most participants preferred high control over their decision (52%) but lower than previous research [10,36]. Decisional conflict levels were low (mean: 22.2 out of 100), which might have been due to participants feeling informed at the time of the questionnaire completion (within 1 week post-delivery of results). Low decisional conflict levels were found in other studies [37]; however, compared with our previous work [10], decisional conflict levels in this study were in a similar range. Future studies could assess whether the low levels of distress, preferred control over decision-making, and decisional conflict associated with QPL usage and high levels of satisfaction with the QPL in our study translate into a better quality of life post-treatment.

This study had several limitations, including the small number of participating recruitment sites, urologists and patients, and the sample size imbalance between the groups (diagnosed vs. not diagnosed with LPC). The large number of participants who did not provide the specific questions they selected means we cannot be sure that our findings regarding the most commonly selected questions are generalisable. Moreover, our sample does not represent the prostate cancer population in general, as the treatment pathway for prostate cancer in New South Wales, Australia, where the study was conducted may differ from other jurisdictions. While we cannot draw conclusions regarding the universal acceptability and feasibility of the QPL, our findings suggested that while the QPL seems to be acceptable to men with suspected prostate cancer, its feasibility as a decision-making tool was limited by it only being provided to 18.4% of eligible men. These findings provide valuable guidance regarding the need for future research focused on strategies to facilitate the uptake of the QPL in clinical practice.

## 5. Conclusions

This study developed a novel, simple and inexpensive tool to prepare men with suspected localised prostate cancer to ask questions about their biopsy results. Our results suggest that the QPL is safe and acceptable, but further work is needed to improve its feasibility. Delivering the QPL before the prostate biopsy results did not increase distress levels. Engagement with clinicians to guarantee that the QPL is offered with the minimum additional workload is critical.

## Figures and Tables

**Table 1:** Participant demographic characteristics.

Characteristic	Diagnosed with LPC (n = 25)	Not Diagnosed with LPC (n = 9)	Total (n = 34)
Age, years, mean (SD) {range} *	65 (8.2) {43–78}	63 (5.6) {51–69}	64.6 (7.7) {43–78}
Highest completed education (%)			
Primary/secondary school	9 (36)	2 (22.2)	11 (32.4)
High school	-	1 (11.1)	1 (2.9)
Post-school vocational	12 (48)	4 (44.4)	16 (47.1)
University	4 (16)	1 (11.1)	5 (14.7)
Higher degree (postgraduate)	-	1 (11.1)	1 (2.9)
Employment status (%)			
Full-time employed	11 (44)	3 (33.3)	14 (41.2)
Part-time employed	2 (8)	1 (11.1)	3 (8.8)
Unemployed	1 (4)	1 (11.1)	2 (5.9)
Retired/pensioner	11 (44)	4 (44.4)	15 (44.1)
Relationship status (%)			
Never married	1 (4)	-	1 (2.9)
Married or de facto	21 (84)	7 (77.8)	28 (82.4)
Widowed		1 (11.1)	1 (2.9)
Divorced/separated	2 (8)	1 (11.1)	3 (8.8)
Unknown	1 (4)	-	1 (2.9)
Country of origin (%)			
Australia	21 (84)	6 (66.7)	27 (79.4)
Other **	4 (16)	3 (33.3)	7 (20.6)
Previous history of cancer (%)			
Yes ^¶^	4 (16)	1 (11.1)	5 (14.7)
No	21 (84)	8 (88.9)	29 (85.3)

LPC, localised prostate cancer. * One participant did not provide this data. ** Chile, Croatia, England, Iraq, Laos, Peru and South Africa (n = 1). ^¶^ Skin cancer (n = 2), genitourinary (n = 2) and lung (n = 1). Note 1: no statistical difference in age between patients with or without localised prostate cancer (*p* = 0.295, t = 0.661).

**Table 2:** QPL usage.

Usage Item	Diagnosed with LPC (n = 25)	Not Diagnosed with LPC (n = 9)	Total (n = 34)	*p*-Value
Format preference				
Printed	21 (84)	7 (77.8)	28 (82.4)	0.59 *
Online	3 (12)	2 (22.2)	5 (14.7)	
Unknown	1 (4)	-	1 (2.9)	
Looked at QPL before biopsy results consultation?				
Yes	17 (68)	9 (100)	26 (76.5)	0.15 *
No	7 (28)	-	7 (20.6)	
Unknown	1 (4)	-	1 (2.9)	
Time spent looking at QPL in minutes (SD) {range}	17.6 (14.3) {2–60}	20 (13.9) {5–45}	18.4 (14) {2–60}	0.49 **
Looked at QPL with someone else				
Yes	7 (28)	4 (44)	11 (32.4)	0.84 *
No	14 (56)	5 (56)	19 (55.9)	
Not applicable (did not use QPL)	2 (8)	-	2 (5.9)	
Unknown	2 (8)	-	2 (5.9)	
If yes, who did you look at it with (e.g., partner, child, friend)				
Partner	6 (85.7)	-	6 (54.5)	
Friend	-	1 (25)	1 (9.1)	
Nurse	-	1 (25)	1 (9.1)	
Unknown	1 (14.3)	2 (50)	3 (27.3)	
Number of selected questions (SD)	8.8 (7.5)	4.75 (4.1)	7.4 (6.7)	**0.02** **
QPL triggered distress				
Yes	1 (4)	-	1 (3)	1 *
No	24 (96)	9 (100)	33 (97)	
Took QPL to the consultation				
Yes	11 (44)	6 (67)	17 (50)	1 *
No	8 (32)	3 (33)	11 (32.4)	
Not applicable (did not use QPL)	3 (12)	-	3 (8.8)	
Unknown	3 (12)	-	3 (8.8)	
Referred to the QPL during consultation				
Yes	10 (40)	4 (44)	14 (41.2)	1 *
No	11 (44)	5 (56)	16 (47.1)	
Not applicable (did not use QPL)	2 (8)	-	2 (5.9)	
Unknown	2 (8)	-	2 (5.9)	
QPL questions asked	5.5 (5.7)	2.75 (1.9)	4.7 (5)	0.12 **

LPC: localised prostate cancer. QPL: question prompt list. Note 1: *p*-value relates to tests of the difference between those diagnosed with LPC vs. those who were not diagnosed with LPC. * Fisher’s exact test. Excluding not applicable or unknown cases. ** Independent *t*-test.

**Table 3:** QPL usefulness and satisfaction.

Item	Diagnosed with LPC (n = 25)	Not Diagnosed with LPC (n = 9)	Total (n = 34)	*p*-Value
How useful was the question prompt list in guiding the consultation with your specialist/urologist?				
Extremely useful/very useful	9 (36)	5 (55.6)	14 (41.2)	0.22
Moderately useful	8 (32)	-	8 (23.5)	
Somewhat useful	2 (8)	2 (22.2)	4 (11.8)	
Not at all useful	1 (4)	-	1 (2.9)	
Not applicable (did not use the question prompt list) or missing	5 (20)	2 (22.2)	6 (20.6)	
How useful was the question prompt list in helping you understand your diagnosis of prostate cancer?				
Extremely useful/very useful	11 (44)	NA	11 (44)	NA
Moderately useful	5 (20)	NA	5 (20)	
Somewhat useful	4 (16)	NA	4 (16)	
Not at all useful	-	-	-	
Not applicable (did not use the question prompt list)	5 (20)	3 (33.3)	8 (23.5)	
How useful was the question prompt list in helping you understand your treatment options for prostate cancer?				
Extremely useful/very useful	12 (48)	4 (44.4)	16 (47.1)	0.590
Moderately useful	4 (16)	-	4 (11.8)	
Somewhat useful	3 (12)	1 (11.1)	4 (11.8)	
Not at all useful	-	-	-	
Not applicable (I did not use the question prompt list)	6 (24)	4 (44.4)	10 (29.4)	
How useful was the question prompt list in guiding your consideration of treatment options?				
Extremely useful/very useful	10 (40)	4 (44.4)	14 (41.2)	0.84
Moderately useful	6 (24)	1 (11.1)	7 (20.6)	
Somewhat useful	1 (4)	1 (11.1)	2 (5.9)	
Not at all useful	1 (4)	-	1 (2.9)	
Not applicable (did not use the question prompt list) or missing	7 (28)	3 (33.3)	10 (29.4)	
Satisfaction with the contents of the question prompt list				
Very satisfied/satisfied	18 (72)	6 (66.7)	24 (70.6)	0.86
Neither satisfied nor dissatisfied	3 (12)	2 (22.2)	5 (14.7)	
Very dissatisfied/dissatisfied	1 (4)	-	1 (2.9)	
Unknown	3 (12)	1 (11.1)	4 (11.8)	
Satisfaction with the layout of the question prompt list				
Very satisfied/satisfied	17 (68)	6 (66.7)	22 (67.6)	0.75
Neither satisfied nor dissatisfied	2 (8)	2 (22.2)	4 (11.8)	
Very dissatisfied/dissatisfied	2 (8)	-	2 (5.9)	
Unknown	4 (16)	1 (11.1)	5 (14.7)	
Satisfaction with the length of the question prompt list				
Very satisfied/satisfied	17 (68)	6 (66.7)	22 (67.6)	0.70
Neither satisfied nor dissatisfied	2 (8)	2 (22.2)	4 (11.8)	
Very dissatisfied/dissatisfied	1 (4)	-	1 (2.9)	
Unknown	5 (20)	1 (11.1)	6 (17.6)	

Note 1: Fisher’s exact test was utilised.

**Table 4:** Decisional outcomes.

Item	Diagnosed with LPC n (%)
Preferred treatment (n = 25)	
Radiotherapy	9 (36)
Robotic prostatectomy	6 (24)
Not sure yet	4 (16)
Active surveillance	4 (16)
Open surgery	1 (4)
Unknown	1 (4)
Control preference: desired involvement in decision-making (n = 25)	
High control	13 (52)
Shared control	7 (28)
Low control	3 (12)
Unknown	2 (8)
Decisional Conflict Scale (n = 24 *)	Mean (SD) {Range}
Total score	22.2 (16.2) {0–60.9}
Uncertainty subscale	27.4 (21.5) {0–83.3}
Informed subscale	17.7 (19.1) {0–75}
Values clarity subscale	23.3 (19.2) {0–75}
Support subscale	20.1 (17.2) {0–66.7}
Effective decision subscale	22.4 (17.4) {0–68.8}

* One participant did not complete multiple items of the scale, and hence, they were excluded.

**Figure A1:**
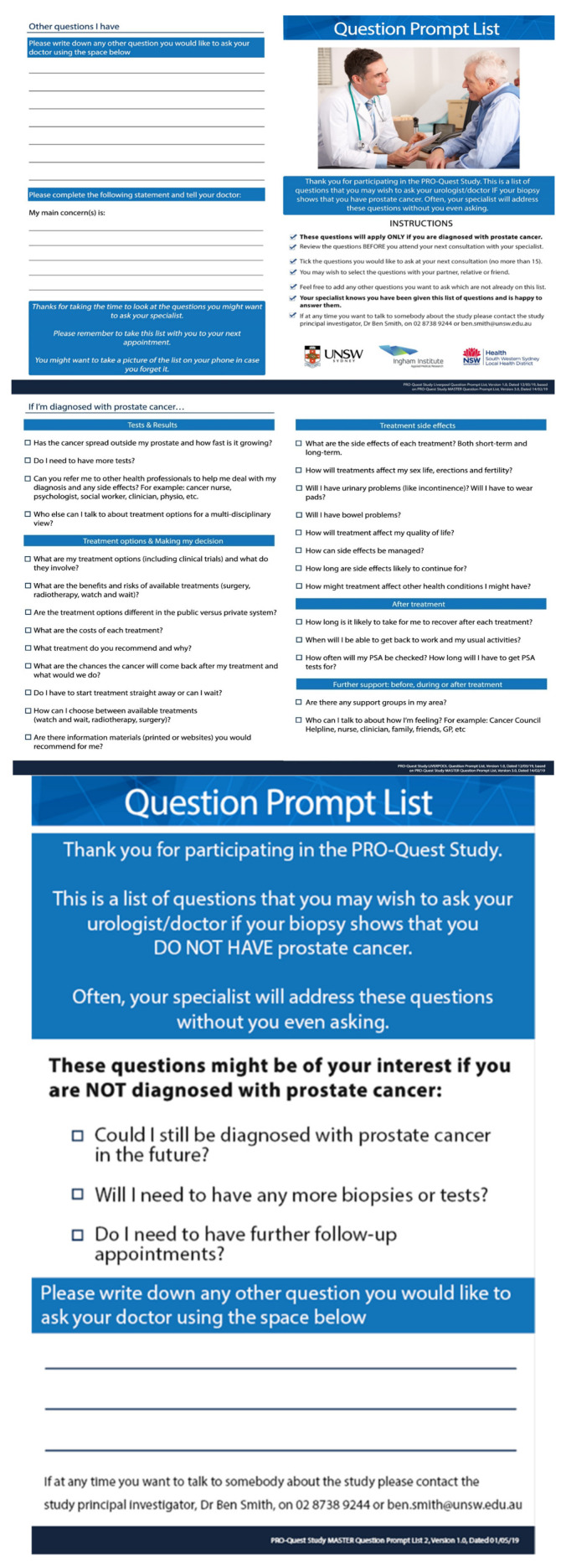
Question prompt list (external and internal view, and additional 1-page brochure).

## Data Availability

The data presented in this study are available on request from the corresponding author. The data are not publicly available due to security, privacy and confidentiality considerations stipulated by the South Western Sydney Local Health District Human Research Ethics Committee.

## Supplementary Materials

The following supporting information can be downloaded at: https://www.mdpi.com/article/10.3390/curroncol30020162/s1, Table S1: Subthemes and additional supportive quotes

## Appendix A

### Appendix A.1. Patient Interview Schedule

#### Appendix A.1.1. General Questions

*Being provided the QPL*
How did you feel about being given the QPL when you had your prostate biopsy?

- Anxious? Overwhelmed? Prepared?

Is there another time you feel it would be better to be given the QPL?

- A bit later? Just before the consultation time while waiting?

*Format of the QPL*
What version of the QPL you selected? Printed or online?
Why did you prefer this format?

#### Appendix A.1.2. Usage/Non-Usage and Satisfaction with the QPL

*QPL usage before the consultation with urologist—General*
Did you have a look to the QPL before your consultation with the urologist?

- Had you checked it before the research team member called 1-2 days before the consultation with your urologist?

If QPL was used before consultation, how much time did you spend looking at the QPL?

- Did you pick many or just a few questions and why?

Did you feel stressed or anxious about using the QPL?

- Can you please explain further?

*For people who looked at the QPL*
What did you like about the QPL? What didn’t you like about the QPL?
What did you think about the questions included in the QPL?

- How understandable were they?
- Were there too many / not enough?
- Were there any questions not listed you think should have been?

What did you think about the layout of the QPL?

- Colours, font, structure.
- How could it be improved?

Are there any changes you think should be made to the QPL?
Did you go through the QPL with anybody else? For example a friend or family member?
*For people who did not take the QPL to their subsequent consultation with their urologist*
What factors stopped you from taking the QPL to your consultation?

- Forgot it, didn’t have time to look at it, no questions, urologist didn’t seem keen on it

For people who did take the QPL to their subsequent consultation with their urologist
Do you feel your urologist encouraged or supported your use of the QPL?
What factors encouraged you to take the QPL to the consultation at which you got your biopsy results?
How many questions did you ask from the QPL?

- What were they about?

Which one was the most important question you wanted to ask and why?
How did your use of the QPL impact on your interaction with your urologist?

- Were they happy to answer questions?
- Were you given enough time to ask questions?
- Were you satisfied with the answers you got to your questions?

How do you think using the QPL impacted on your treatment decision-making?

- Did you ask any questions?
- Did you ask about or consider any treatment options other than surgery?
- Did you consider seeing another specialist or getting a second opinion?

Would you recommend the QPL to a friend in future?

- Why? Why not?

## Appendix C

**Table A1:** Most frequently selected questions.

Question	Number of Times Selected	% *
1. Has the cancer spread outside my prostate and how fast is it growing?	10	76.9
2. Do I need to have more tests?	4	30.8
3. Can you refer me to other health professionals to help me deal with my diagnosis and any side effects? For example: cancer nurse, psychologist, social worker, clinician, physio, etc.	1	7.7
4. Who else can I talk to about treatment options for a multidisciplinary view?	2	15.4
5. What are my treatment options (including clinical trials) and what do they involve?	4	30.8
6. What are the benefits and risks of available treatments (surgery, radiotherapy, watch and wait)?	5	38.5
7. Are the treatment options different in the public versus private system?	2	15.4
8. What are the costs of each treatment?	3	23.1
9. What treatment do you recommend and why?	8	61.5
10. What are the chances the cancer will come back after my treatment and what would we do?	5	38.5
11. Do I have to start treatment straight away or can I wait?	4	30.8
12. How can I choose between available treatments (watch and wait, radiotherapy, surgery)?	3	23.1
13. Are there information materials (printed or websites) you would recommend for me?	2	15.4
14. What are the side effects of each treatment? Both short-term and long-term.	10	76.9
15. How will treatments affect my sex life, erections and fertility?	4	30.8
16. Will I have urinary problems (like incontinence)? Will I have to wear pads?	7	53.8
17. Will I have bowel problems?	6	46.2
18. How will treatment affect my quality of life?	7	53.8
19. How can side effects be managed?	4	30.8
20. How long are side effects likely to continue for?	6	46.2
21. How might treatment affect other health conditions I might have?	2	15.4
22. How long is it likely to take for me to recover after each treatment?	8	61.5
23. When will I be able to get back to work and my usual activities?	5	38.5
24. How often will my PSA be checked? How long will I have to get PSA tests for?	3	23.1
25. Are there any support groups in my area?		0.0
26. Who can I talk to about how I’m feeling? For example: Cancer Council Helpline, nurse, clinician, family, friends, GP, etc	2	15.4
**Additional 1-page QPL (scenario NOT diagnosed with LPC)**		
Could I still be diagnosed with prostate cancer in the future?	1	7.7
Will I need to have more biopsies or tests?	2	15.4
Do I need to have further follow-up appointments?	1	7.7

***** Out of 14 participants; PSA: prostate-specific antigen; GP, general practitioner; LPC: localised prostate cancer.
