# Supplementary material for: Encouraging Patients to Ask Questions: Development and Pilot Testing of a Question Prompt List for Patients Undergoing a Biopsy for Suspected Prostate Cancer
Source: Curr Oncol. 2023 Feb 8;30(2):2088–104. doi: 10.3390/curroncol30020162 (PMC9954987; doi:10.3390/curroncol30020162)
Supplement: Supplementary file 1 [file curroncol-30-00162-s001.zip › curroncol-2128448-supplementary.pdf]

Supplementary material

Table S1. Subthemes and additional supportive quotes

Theme	Subtheme & description	Supportive quotes
The freedom to ask: acceptable timing, flexible usage and usefulness of the QPL	*Acceptable delivery time with no additional distress:* most participants considered that the QPL was delivered at the appropriate time and did not express increased anxiety or distress due to the QPL. A few participants thought that the QPL could be given at the very first consultation with their urologist (before biopsy), while a few others believed it could be given after biopsy results.	I think it's the perfect time to send it out. P040<br>Well, I felt good because you had it before your diagnosis. P001<br><br>…you could probably go through it [QPL] and you could already ask him other questions that are on the flyer before you actually go back to him to get your biopsy results. You could already ask him those questions from the very first consultation… So, if you had this before you went to your biopsy results and said, "If my biopsy results aren't good, what's the likelihood of me – what should I expect?" So instead of waiting for your biopsy results to come back or even waiting to go to have your biopsy, 'cause I waited two weeks, you could already have had them being answered. P003<br><br>Maybe after the biopsy and then once you know whether you are positive or not, maybe then. Instead of worrying about it before you have the biopsy, you'd ask all these questions and there's a lot of doubt in your mind, you're wondering if this happens or if that happens, and it just gives you that much longer to wait, I think, and wonder. P035<br><br>No problem at all. It just gave me a few ideas, what to ask. I didn't feel anxious or worried at all. I was quite comfortable with it. I didn't have an issue at all. No problems at all. P008<br><br>I didn't feel stressed at all. P040

	Flexible usage: high engagement with the QPL before appointment: the majority of participants looked at the QPL before the appointment to receive the biopsy results. Generally, they looked at the QPL as soon as they received it and before the reminder made 2-3 days before the appointment with their doctor. Only one participant indicated he did not look at the QPL due to having a busy lifestyle. Most participants looked at the QPL multiple times. Some looked at it with a family member (generally a partner) or a friend, which triggered a conversation about the topics pointed out by the QPL. Others looked at it individually. Most participants reported during interviews spending around 10 minutes looking at the QPL; one participant only scanned it briefly, while two participants took around 1 hour looking at the QPL and discussing it with a family member. The selection of questions was quite diverse and ranged from not picking any questions to picking most of them before the consultation.	…when I went back in my second appointment, my wife come to and we took the flyer with us and we ticked off what we wanna ask and all that. It was nearly every question that you had on the flyer, we ticked but when Dr [urologist surname] started talking. He was answering question before we even asked. P001<br><br>…it would've been half an hour one day [looking at the QPL], and a few minutes the next day… It didn't take it long to get through it. P008<br><br>…there were some days where I'd spend 10 or 15 minutes and put it [the QPL] down and I'd go and do whatever I had to do, and I might come back at lunchtime and I know is gonna be there and I'd pick it [the QPL] up and go over it again and have a bit of a look. It was – I went over it quite a bit… it'd be three or four times [looking at the QPL]… I picked a few of them [questions]. P003<br><br>…my wife looked at it [QPL]… we ticked nearly all of them [questions] actually. She went through all those questions… P001<br><br>I didn't really go through the flyer that totally, to be honest. Okay, I just sort of – I had skimmed through it… P047<br><br>I'd say it was about around an hour I took to go through the flyer. P004<br><br>I just shared it with my wife… We discussed it [QPL] for maybe

		an hour or so, from memory, yes. P035
	QPL as an information-seeking and confidence facilitator: the QPL encouraged participants to ask the questions that mattered the most to them and ask more questions than they originally had thought. The QPL also motivated them to look for information before the consultation where biopsy results are provided. The QPL triggered curiosity about the different facets of prostate cancer and helped at framing the questions they wanted to ask. Some participants expressed that the QPL provided them with reassurance, understanding about what to expect from the consultation, and confidence about addressing some topics during the encounter with their doctor. One participant noted feeling more prepared for a second consultation after biopsy results were delivered.	I found it quite helpful in making sure that I knew what I should ask the doctor. P004<br><br>I feel that it's rather helpful. When I looked at it and ran through it with a bit more time, I liked it because there are things that an old person like me wouldn't think of or saying or asking. It gave me a bit more knowledge about what problems that I should ask about… It's very helpful. P008<br><br>Well, the flyer gave me the opportunity or the catalyst to ask questions. It said to me it's okay to ask anything, basically… reading the flyer gives you the thought that these are things that you should be asking, not just sitting there staring into space sort of thing. So that encouraged me to ask questions. P016<br><br>I still found that it prompted me to ask maybe a couple more questions and to reword how I'd ask them. P004<br><br>We used the flyer [to seek information]. And my partner, she looked it up on the Google and then went back to the flyer and we looked around and searched... P027<br><br>It's very handy… it would really give you a little bit of help and maybe a little bit more confidence about what to say, how to act and what to ask the specialist. P008<br><br>It's like a reassurance sort of thing, so you know what to expect and what you're talking

		about when you go and ask him these questions. Otherwise, you don't think, "What's gonna happen after the diagnosis," things like that. P001<br><br>At least they [urologists] give you something [the QPL] to try and put your mind at rest… it's not something that's big and bulky, it's just a basic piece of information so that it's not overwhelming. It's enough to at least get you started … P003
	The unchanged consultation dynamic and decision-making process: when it comes to the use of the QPL during the consultation, most participants indicated they did not use it during the actual encounter with their doctor. Only a few participants mentioned that they took the QPL with them and used it during the consultation. However, some of those who did not use the QPL during the consultation suggested that they remembered questions from it and asked them to their doctor. Another set of participants noted they did not ask any questions, which might be related with the fact that most participants were satisfied with their urologist who naturally addressed the key topics and questions without being asked. Participants felt they had enough time to ask questions, their doctors were happy to answer questions. Participants expressed satisfaction with the responses from their urologists. Moreover, the QPL did not seem to have had an impact on treatment decision-making. A few participants revealed a passive role in their decision-making process, while one participant mentioned that the QPL broadened his treatment choices.	Unchanged consultation dynamic<br><br>… and when we got to Dr [urologist's surname], I said, "I hope you don't mind I've got these questions I wanna ask," and then he started talking, and she [wife] said, "Oh well, there's not more we can ask you [to the urologist] 'cause you've answered nearly every question," P001<br><br>But as I said, going through the list at the consultation, Dr [urologist surname] had answered most of those questions before I even had a chance to ask them. P026<br><br>…I didn't end up really using it [the QPL]… If tested negative, I think I would've thought bring it out then but being overwhelmed with the diagnosis, there was all too much going on. P008<br><br>I had it [the QPL] in my pocket and then towards the end of the consultation, I briefly looked at it and said, "Yep, I've asked what questions I wanted to ask and I've been given the answers very clearly from the doctor." P004

		Unchanged decision-making process<br><br>I don't remember the prompt list actually convinced me to go this way [having surgery]. P003<br><br>…It [the QPL] didn't make an impact [on treatment decision]. I thought really the two doctors I've seen were fine… So talking to both Dr [urologist name] and the doctor who's in Campbelltown Hospital [radiation oncologist] had helped really make my decision easy. P028<br><br>I didn't know I had any options [treatment options], mate. I was up – you know what I mean? I just thought that whatever the doctor said I would have to accept that. But I realise now that there are many different options, mate, that I can take. P040
Satisfaction with the QPL content	*Satisfaction with the comprehensiveness and comprehensibility of the QPL:* participants found that the number of questions was acceptable and did not require a very long time to review; they did not feel that questions were overwhelming or thought of any significant questions being missed. One participant indicated that the questions were very generic, and he had more specific questions to ask. They agreed that the questions were easy to understand.	They were quite easy to understand. P027<br>They're very easy and straightforward. I had no issues with that at all. It was extremely easy to work with. P008<br><br>I think it covers it [the QPL] all pretty well… you need to have all the basic ones [questions] like the procedures, going through the risks and how is your life gonna be after, and all those things. I think they're important questions, just a quick through. P003<br><br>They [the questions] were quite straightforward. There wasn't any medical term type stuff in there that can often throw you off… But now I thought it was in a good plain English sort of

		thing that it was quite easy to understand. P026
	User-friendly structure: participants noted that the layout and structure of the QPL were simple and easy to follow. Positive comments were expressed about the QPL size, font and colours. No changes were suggested to the layout and structure of the QPL.	… it's pretty well-explained with the test and results and then the treatment options and making decisions and side effects. The layout is – you couldn't really ask for a better layout. P004<br><br>I think the layout or the structure was pretty good. It looks really good. I don't know what you could've done with it. P028<br><br>…it was easy to use and – yeah, user friendly. P026
	QPL potential improvements: A few participants offered suggestions about some elements that could be added to the QPL, including the addition of a) links to support groups or reputable websites to obtain information about prostate cancer, b) brief information about cancer risks or information to reassure patients, c) questions about the meaning of the Gleason score, prevention, healthy lifestyles, and more post-treatment details.	So I think that maybe in your flyer, you have links to support groups or different information from people that are actually patients. P016<br><br>The Gleason score. Yeah. Because Dr [X] said, "In Australia, we don't use that." He said, "I don't put a lot of reliance in that score." He said, "'Cause they don't even start 'til six." He sort of brushed over that. But I spoke to my GP last week and asked him about it. He explained it all, checked what it all meant, everything like that. So I understood that a bit better. P026
